# Poly(lactic acid)–Poly(butylene succinate)–Sugar Beet Pulp Composites; Part II: Water Absorption Characteristics with Fine and Coarse Sugar Beet Pulp Particles; A Phenomenological Investigation

**DOI:** 10.3390/polym13203558

**Published:** 2021-10-15

**Authors:** Rodion Kopitzky

**Affiliations:** Department of Circular and Bio-based Plastics, Fraunhofer UMSICHT, Fraunhofer Institute for Environmental, Safety and Energy Technology, Osterfelder Str. 3, 46047 Oberhausen, Germany; rodion.kopitzky@umsicht.fraunhofer.de

**Keywords:** sugar beet pulp, composites, bio-based polymers, water absorption, diffusion, relaxation, simulation

## Abstract

Sugar beet pulp (SBP) is a residue available in large quantities from the sugar industry, and can serve as a cost-effective bio-based and biodegradable filler for fully bio-based compounds containing bio-based polyesters. The composition of SBP is characterized by an unusually high content of pectins, which are known as water-binding substances. Their molecular structure and the poor gelling properties, compared to other pectin sources, do not allow industrial use on a larger scale. However, good water absorption capacity can be advantageous for promoting plastics degradation or disintegration in the environment. In this study, we evaluated the water absorption capacity and processes of SBP-filled composites with bio-based polyesters on a longer time scale. We analyzed water absorption from a phenomenological point of view and tried to derive basic parameters for the general description of the composites behavior. We found that polar polyesters or polyester blends filled with higher amounts of especially coarse SBP suffer disintegration within a few weeks when supplied with sufficient water. On the other hand, less polar polyesters filled with fine SBP rather absorb water but do not show disintegration for several months. On a time scale of a few years, catalytic disintegration of the composites appears to be independent of the addition of SBP.

## 1. Introduction

Sugar beet is one of the most productive crops of the world. According to the data of the Food and Agriculture Organization of the United Nations, the harvested amount adds up to approximately 308 million tons in 2019, of which about half comes from Europe and one-third from the former Russian Federation [1]. The water insoluble residue after extraction of the sugar, the sugar beet pulp (SBP), consists mainly of approximately equal parts of cellulose, hemicellulose and pectins (in sum 75–85%) and smaller amounts of lignin (<9%), proteins (<7%), lipids (<2%), saponins (<2%) and ash. Approximately 45–50 kg of sugar beet pulp (dry matter) can be obtained from 1 ton of fresh sugar beet [2,3,4].

In our recent paper [5] we summarized the literature of SBP containing bio-based and/or biodegradable composites, mainly from the last two decades and reported our work on material characterization of PLA–PBS–SBP composites. According to this, SBP containing composites represent a material with expectable mechanical properties. Due to the different polarities of the polymeric components and the highly polar SBP, compatibilizers have to be used to enable higher moduli and strengths. However, the maximum achievable properties are limited due to the low tensile modulus of approx. 5000 to 7000 MPa [5,6] acting in the composites. Particularly in the case of coarse SBP particles, which may consist of cell agglomerates due to the cell structure of the sugar beet plant and therefore have a low inherent strength, the strength of such composites are limited. Even high amounts of compatibilizers cannot further increase their strength. In addition, SBP containing composites often exhibit small elongation at break of only a few percent and impact strengths in the range of 1 to 3 kilojoules per square meter. We therefore concluded that biodegradable and full or partially biobased PLA–PBS–SBP composites are, from a material mechanics point of view, just one of many composites with agricultural residues as filler, and therefore do not represent anything unique to pay special attention to.

However, in contrast to classical natural fibers, SBP has the peculiarity of high content of pectin. SBP, such as thermoplasticized starch, can be processed with plasticizer in an extrusion cooking process to produce a pure SBP plastic material [2,7,8,9]. The plasticized pectinic phase therein acts as the matrix for the non-soluble components of SBP. From a materials point of view, however, such pure thermoplasticized SBP materials suffer from their high sensitivity to water [9].

Even though the quality of sugar beet pectins does not approach that of apple and citrus pectins concerning water uptake and gelation [10,11], they are produced in low amounts with some niche applications [12,13]. Nevertheless, SBP pectins can form gels if the disturbance of the gelling ability, which is caused by the acetylation [10,14,15,16] of the secondary hydroxyl groups, is forced by deacetylation or chemical crosslinking [17]. Due to the extraction and purification processes, pure pectins are expensive chemicals. However, even without any chemical conversion, composites with SBP as filler will be highly sensitive to water [18].

Fully biodegradable composites may play a role in end-of-life options for application in agriculture and horticulture beside the well-known mulch films. Due to their thinness, biodegradation will take place in shorter time periods than for products with dimensions in the millimeter range or beyond. Such thick-walled products often suffer from their low surface to volume ratio and the limited attack of the microbiome to the surface. On the other hand, the possible degradation of polyester by hydrolysis is very slow under environmental conditions due to low water absorption and lack of catalytic support in the bulk material if proton and water concentrations are low [19]. Composites containing a strongly water-attracting and water-retaining material can therefore support the internal degradation processes. Additionally, they may have a destructive effect through relaxation processes as a result of water absorption. In this article, we report our phenomenological investigation on the water absorption of PLA–PBS–SBP composites.

## 2. Materials and Methods

### 2.1. Materials

Polymers: Poly (lactic acid) (Ingeo 3251D, NatureWorks LLC, Minnetonka, MN, USA) was purchased from Resinex Germany GmbH (Zwingenberg, Germany). Poly (butylene succinate) (injection molding grade) was a donation from MCPP Germany GmbH (Düsseldorf, Germany).

Fillers: Chalk was purchased from Omya GmbH (Cologne, Germany). Talc was purchased from Mondo Minerals B.V. (Amsterdam, The Netherlands).

Additives: Maleic anhydride modified PLA (PLA-g-MAH, development type) was a donation of BYK-Chemie GmbH (Schkopau, Germany). All chemicals were used as received.

Ground SBP types were provided by Pfeifer & Langen (Cologne/Elsdorf, Germany) in paper bags. The grinding of the SBP had been accomplished before by Jäckering Mühlen- und Nährmittelwerke GmbH (Hamm, Germany) using an air turbulence mill of type “Ultra-Rotor”. Two ground types were used. A fine type (D50 = 23.4 µm), in which the cell structure is destroyed and only cell wall remnants are present, and a coarse type (D50 = 500/600 µm, two different lots), in which intact cells still exist. Pictures of the cell structure and ground SBP are presented in our previous publication [5].

We analyzed the water content of the SBP prior to use and noticed that it changed from about 8 to 9 percent after receiving the sample to about 10 to 11 percent before processing depending on humidity conditions. Compound compositions were calculated on a dry mass basis. Due to the natural origin of SBP, deviations in the content of its constituents may occur using SBP from different years and/or different cultivation areas. No analysis of the SBP components was performed.

### 2.2. Compounding

An intermeshing co-rotating twin-screw extruder ZSK 25 from Coperion (Stuttgart, Germany) with the screw diameter D being 25 mm and the screw length L = 40 D was used for compounding. The polymers were dry blended and fed by a gravimetrical dosing feeder into the hopper. SBP was dry blended with coupling agent and/or fillers and fed by a gravimetrical dosing feeder into a side feeder.

The extruder consists of eight zones, which can be tempered individually. In the area of the second zone, a liquid dosing connection is installed. A side feeder for the addition of further materials (SBP, filler, coupling agents) follows this. This zone is also equipped with an atmospheric degassing system. At the beginning of the seventh zone, volatile components can be removed from the melt with the aid of a vacuum suction port (~200 mbar). A nozzle plate with two 3 mm diameter nozzle holes completed the process. Details of screw design are described in our previous work [5] (screw design 2).

Zone temperatures of the extruder were set to 60, 170, 170, 170, 170, 160, 160 and 165 °C for the production of SBP composites with PLA/PBS. The measured temperature of the materials at the die was 176 ± 3 °C (1σ). The strands were water cooled, granulated by an SGS 50-E granulator (Reduction Engineering GmbH Korntal-Münchingen, Germany) and dried in a dryer (dry air generator, model: LUXOR 80; drying bins: 15 L, Motan-Colortronic GmbH, Kirchlengern, Germany) at 60 °C for several hours until the humidity was below 0.02% (moisture balance MA 30, Sartorius AG, Göttingen, Germany).

Compounds with PLA, PBS and SBP were produced in different grades. As starting formulations, we selected compounds based on three different PLA:PBS ratios with mineral fillers and replaced the mineral fillers with SBP in three or two steps [5]. For improving the adhesion of the SBP to the matrix, PLA-g-MAH was used as coupling agent. The compositions and the alphanumeric codes for sample designation are shown in Table 1:

### 2.3. Injection Molding

Test specimens were injection molded on a Battenfeld 600 injection molding machine equipped with a standard injection molding tool according to ISO 20753:2017 Type 1A (ISO 527 dog-bone-shaped, 2 nests). Die temperature: 170 °C; shot volume: 36–37 cm^3^; injection speed: PLA rich blends: 10 cm^3^ s^−1^, for PBS rich blends a 4-step profile with higher velocities was used; holding pressure: 700–800 bar depending on composition; holding time 20 s (30 s PBS rich); cooling temperature and time: 30 °C/30 s. The compounds were pre-dried in a dryer (dry air generator, model: LUXOR 80; drying bins: 15 L, Motan GmbH, Germany) at 60 °C for 1.5 h immediately before injection molding.

### 2.4. Microscopy

Optical microscopy was conducted with a Keyence VHX 6000 System (Neu-Isenburg, Germany) equipped with a VH-Z20T objective (20×–200×) and a VHX-S660E specimen stage.

### 2.5. Water Absorption

The specimens for water adsorption tests were equilibrated in air with ambient humidity and not dried prior to measuring. The dog-bone test specimens were immersed in distilled water and stored at 23 ± 3 °C in 2 L boxes. About 15–24 specimens, three of each material composition, were put in one box. We removed the test bars individually, immediately freed them from adhering water with a linen-cotton cloth and weighted them on a 0.1 mg balance. Due to the evaporation of molecular films, which cannot be wiped off from the surface, the balance showed a drift which slowed down. We took the first stable value (~10–30 s). Calculation of water absorption was completed according to Equation (1):

(1) $$\frac{w_{t}-w_{0}}{w_{0}}=relative\;absorption$$

with *w_t_* indicating the weight of the specimen after time t and *w*_0_ indicating its weight prior to water immersion. Arithmetic mean values of the relative absorption values of the three specimens were used.

## 3. Background: Penetrant Sorption in Polymers

Due to better understanding of the sorption curves a short introduction into some fundamentals of sorption into polymers will be given here. The text is based on the review from Bond and Smith [20] as well on the paper of Mensisteri et al. [21] and Petropoulus et al. [22]. The fundamental mathematics of diffusion can be found e.g., in the classical textbook from Cranck [23].

The description of sorption processes of penetrating substances (penetrants) in polymers can ideally be described by assuming a Fickian diffusion profile [23]. The mathematical treatment of the underlying second law of Fick (Equation (2)):

(2) $$\frac{\partial c}{\partial t}=D\frac{\partial^{2}c}{\partial x^{2}}$$

provides in the integrated form a method to determine the diffusion coefficient (Equation (3); *M_t_*, *M_∞_*: mass uptake at time *t* or *∞*, respectively; *l*: half of the thickness of a film or plate of thickness; *D*: diffusion coefficient). At the beginning of the sorption process, this type of diffusion is characterized by a dependence of the absorbed mass *M_t_* on the square root of time (Equation (4), concentration of penetrant at *c*(*x* = ±*l*, *t* = 0) = *c_surface_*; *c*(*x*, *t* = 0) = *c*_0_) [22] and *M_t_/M_∞_* < 0.6 [23]). Additionally, an independence of the specific absorption from sample thickness follows.

(3) $$\frac{M_{t}}{M_{\infty }}=1-\frac{8}{\pi^{2}}\;\overset{\infty }{\underset{n=0}{\sum }}\frac{1}{{\left(2n+1\right)}^{2}}exp\left(\frac{-D\left(2n+1\right)\pi^{2}t}{4l^{2}}\right)$$

(4) $$\frac{M_{t}}{M_{\infty }}=2{\left(\frac{Dt}{\pi l^{2}}\right)}^{0.5}\;if\;\frac{M_{t}}{M_{\infty }}<0.6$$

The characteristic diffusion time of a process is defined by [20,22] (Equation (5)):

(5) $$\frac{l^{2}}{D}=t_{Diff}$$

However, water absorption cannot always be adequately described by Fick’s law of diffusion with constant boundary conditions. Ideal Fickian type sorption occurs when the diffusion rate is much faster than the relaxation rate of the polymer. This often occurs with polymers above the glass point. It is generally assumed that deviations from Fick’s behavior correlate with relaxation processes at limited velocity. Absorption processes are therefore divided into three cases [20,21]:

Case 1 (diffusion-controlled adsorption, low uptake, in general *T* < *T_g_*, and/or low activity of penetrant) or Fickian diffusion, in which the transport of the penetrant is essentially a stochastic diffusion process driven by the presence of a chemical potential (e.g., concentration gradient). Such transports generally take place in polymer penetration systems in which the penetration agent has a negligible hygroelastic effect on the polymer, i.e., the rate of diffusion is significantly higher than the rate of relaxation processes. Case I adsorption is characterized by the dependence of the absorption from the square root of the exposure time from the beginning (and the specific absorption is also independent of the sample thickness, Equation (4)). Diffusion and Relaxation are decoupled.

Case 2 (relaxation-controlled adsorption, high uptake, in general *T* > *T_g_*, *T* < *T_g_* and high activity of penetrant) in which the relaxation of the polymer in response to the osmotic pressure of the penetrant is faster than any internal diffusion process. As a result, the penetrant tends to move into the polymer with an unsteady concentration profile; areas of the swollen, saturated polymer are separated clearly from the unswollen dry polymer. The hygroelastic swelling is the result of the reorganization of the macromolecules of the polymer to reduce the osmotic pressure of the penetrant. Case 2 sorption characteristics is reported for polymers below their glass transition temperature include (1) a stepwise discontinuity of the penetration concentration within the polymer from a glassy region to a plasticized swollen region with a relatively high penetration concentration, and (2) an initial linear progress of the discontinuity with time in plate-shaped samples and thus a linear mass increase with time (see also [24]). The linearity of the mass increase ends with a change of the migration velocity of the swollen polymer-penetration medium front. Diffusion and Relaxation are strongly coupled.

Case 3 non-Fickian or anomalous adsorption as a mixture of cases 1 and 2 in which two sub cases can additionally be distinguished [20,21]: 3a, the relaxation controlled adsorption (*T_g_* > *T* moderate uptake, separate processes of water uptake may be clearly be seen; partial decoupling) and 3b, the diffusion controlled relaxation (*T_g_* < *T*, high uptake, no separate processes, ~linear uptake with the square root of time, *t* > *t*_0_, *M_t_/M_∞_* > 0.6, see Equation (4)).

Real absorption curves often show characteristics of both limit cases or subcases. The sorption mechanism is controlled by the physico-chemical behavior occurring within the polymer or polymer composite system and may include aspects of dissolution processes, interactions between polymer/composite and penetration agent (e.g., hydrogen bonds), diffusion, relaxation, swelling and stress build-up. Thus, unusual diffusion is not necessarily a separate process from the two limiting cases, but a process controlled to varying degrees by the limiting mechanism (diffusion and relaxation) and influenced by the interaction between the penetrant and the absorbent. External factors such as the morphology of the composite, temperature, external loadings and the penetrant activity also influence the absorption mechanism.

A qualitative number for describing the coupling of diffusion and relaxation is the Deborah number “Deb” (Equation (6)) which is the ratio of the characteristic relaxation time *λ* to the characteristic diffusion time of the penetrant molecule [20,22]:

(6) $$Deb=\frac{\lambda_{Relax}}{t_{Diff}}=\frac{D}{\beta l^{2}}$$

(7) $$\frac{\partial c}{\partial t}=\beta \left(c_{\infty }-c_{t}\right)\;↔\;\frac{\partial M}{\partial t}=\beta \left(M_{\infty }-M_{t}\right)$$

(8) $$\frac{M_{t}}{M_{\infty ,R}}=\left(1-e^{-\beta t}\right)$$

The relaxation caused by the penetrant can be described by an exponential approach (Equations (7) and (8)). The actual change in concentration per time of the penetrant is proportional to the difference of finite concentration and actual concentration. The proportional factor is the frequency factor *ß*, which is the inverse of the characteristic time *λ* of the relaxation (*M_∞,R_*: water uptake due to relaxation on infinite time).

Below the glass transition temperature chain mobility is low and the completion of the penetrant induced local relaxation may be sufficiently slow to exercise a substantial, or even controlling, influence on the course of the sorption process [22].

If Deb is much greater than 1 (being *D/l*^2^ or *D* high in relation to *β*), the characteristic time of the relaxation process is much greater than the time associated with the diffusion time and the processes are decoupled. Dependent of the specific times, at first a purely Fickian sorption will be present until a (Fickian type) equilibrium is reached which is then followed by a second sorption process due to relaxation of the polymer. Conversely if Deb is much less than 1 the relaxation process is much faster and diffusion and relaxation act simultaneously and case 2 behavior will be seen (normally if test temperature is higher than glass transition temperature of the polymer). If Deb is around 1 anomalous case 3 diffusion characteristics occur.

The distinction of a coupled diffusion relaxation two-stage sorption, which will be modelled by Equation (9) (Equation (2) extended by an exponential term [20,21]; *M_∞,F,r_/M_∞_* = mass uptake at infinite time belonging to Fickian (F) or the relaxation (R) fraction, resp.):

(9) $$\frac{M_{t}}{M_{\infty }}=\frac{M_{t,F}+M_{t,R}}{M_{\infty ,F}+M_{\infty Rr}}=\frac{M_{\infty ,F}}{M_{\infty }}\left[1-\frac{8}{\pi^{2}}\;\overset{\infty }{\underset{n=0}{\sum }}\frac{1}{{\left(2n+1\right)}^{2}}exp\left(\frac{-D\left(2n+1\right)\pi^{2}t}{4l^{2}}\right)\right]+\frac{M_{\infty ,R}}{M_{\infty }}\left(1-e^{-\beta t}\right)$$

from a system with a polymer penetrant interaction mechanism [20] (also named two stage sorption or pseudo-Fickian uptake) may be not obvious at once: This pseudo-Fickian uptake arises from a second diffusion mode covering special sites in the polymer with other binding properties (Equation (10)).

(10) $$\frac{M_{t}}{M_{\infty }}=\frac{M_{t,1}+M_{t,2}}{M_{\infty ,1}+M_{\infty ,2}}=\overset{2}{\underset{i=1}{\sum }}\frac{M_{\infty ,F,i}}{M_{\infty }}\left[1-\frac{8}{\pi^{2}}\;\overset{\infty }{\underset{n=0}{\sum }}\frac{1}{{\left(2n+1\right)}^{2}}exp\left(\frac{-D_{i}\left(2n+1\right)\pi^{2}t}{4l^{2}}\right)\right]$$

Especially, polar penetrants like water may cover sites resulting from the (apolar) free volume plus special polar sites (polar chain ends, hydrogen bonding capable groups, voids, molecular sized gaps between the phases in composites). The resultant sorption may then be modelled by two Fickian terms with different diffusion coefficients (see [25,26] for example).

In general, the superposition of the separated processes in Equations (9) and (10) may be extended to a generalized Equations (11) and (12):

(11) $$\frac{M_{t}}{M_{\infty }}=\underset{i}{\sum }\frac{M_{\infty ,F,i}}{M_{\infty }}\left[1-\frac{8}{\pi^{2}}\;\overset{\infty }{\underset{n=0}{\sum }}\frac{1}{{\left(2n+1\right)}^{2}}exp\left(\frac{-D_{i}\left(2n+1\right)\pi^{2}t}{4l^{2}}\right)\right]+\underset{j}{\sum }\frac{M_{\infty ,R,j}}{M_{\infty }}\left(1-e^{-\beta_{j}t}\right)$$

(12) $$\underset{i,j}{\sum }\frac{M_{\infty ,Fi,Rj}}{M_{\infty }}=w_{\infty ,Fi,Rj}=1$$

It should be kept in mind that the above listed equations only represent model equations, which may not explain all processes on a molecular level in full detail. Especially, no models for explaining case 2 sorption are given here (see [24,27]) and variations of dimension were not taken into account. The mentioned literature provides further insights in this highly complex theme.

On long time scales we noticed an additional effect in the experiments (see Section 4.1 and Section 4.2). It is an accelerated water absorption with time, which is particularly pronounced in SBP free composites. This accelerated water absorption is caused by the onset of hydrolysis of the polyesters, described by the following kinetic description (Equation (13)):

(13) $$\frac{\partial {\left[H_{2}O\right]}_{uptake}}{\partial t}=k\left[Ester\right]\left[Acid\right]{\left[H_{2}O\right]}_{free,a}$$

[*H*_2_*O*]*_free,a_* is the free water in the composite with activity a, which is not bound to strong sorption sites (e.g., the pectinic acid groups or terminal acid groups of the polyesters) and which can react with the ester functions. Assuming that this amount of reactive water is constant, also assuming that the amount of ester bonds is nearly constant in the time range in which the composites do not show mechanical failure, and taking in mind that the change in water uptake is proportional to the change in newly generated acid groups by hydrolysis, Equation (13) can be rewritten:

(14) $$\frac{\partial \left[Acid\right]}{\partial t}=k^{\prime }\left[Ester\right]\left[Acid\right]{\left[H_{2}O\right]}_{free,a}=k^{\prime \prime }\left[Acid\right]$$

(15) $${\left[Acid\right]}_{t}={\left[Acid\right]}_{o}e^{k^{\prime \prime }t}\;↔\;{\left[H_{2}O\right]}_{uptake,t}={\left[H_{2}O\right]}_{uptake,0}e^{\beta t}$$

Equation (11) must therefore be extended by a further exponential element and partial weight fraction:

(16) $$\frac{M_{t}}{M_{\infty }}=\underset{i}{\sum }\frac{M_{\infty ,F,i}}{M_{\infty }}\left[1-\frac{8}{\pi^{2}}\;\overset{\infty }{\underset{n=0}{\sum }}\frac{1}{{\left(2n+1\right)}^{2}}exp\left(\frac{-D_{i}\left(2n+1\right)\pi^{2}t}{4l^{2}}\right)\right]+\underset{j}{\sum }\frac{M_{\infty ,R,j}}{M_{\infty }}\left(1-e^{-\beta_{j}t}\right)+\frac{M_{t,expo.}}{M_{\infty }}\left(e^{\beta_{expo.}t}\right)$$

As this exponential element has no finite value, the partial weight fractions (Equation (12)) are dependent from the time (saturation level *M_∞_*) and the partial weight fractions for different compositions of the composites are not comparable directly. Absolute values (see Equation (12): *w_∞ Fi,Rj_ × M_∞_ = M_∞,Fi,Rj_*) for each characteristic step of adsorption should be chosen. When comparing composites with different amounts of SBP, the absolutes values per percent of SBP give comparable values (see Section 4.2).

## 4. Results

### 4.1. Water Sorption in Sugar Beet Pulp Containing Composites

#### 4.1.1. Phenomenological Description

**Figure 1:**
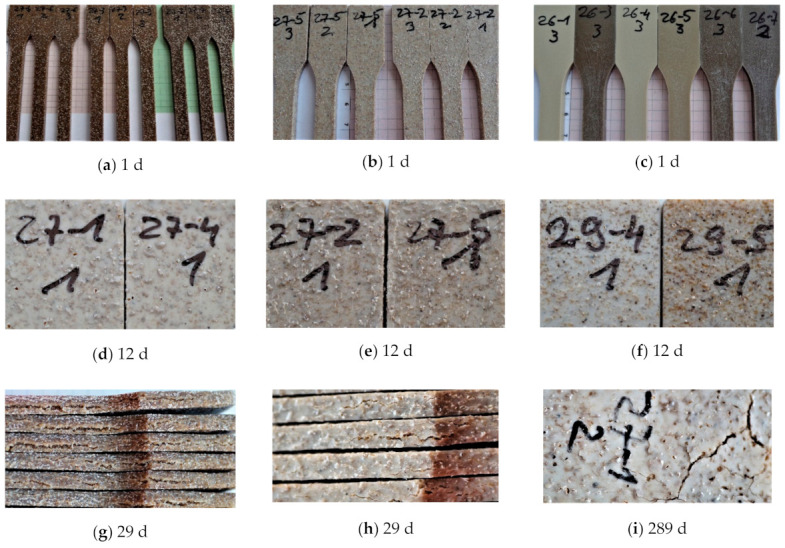
Tensile test bars immersed in water; except (**c**) course SBP type; (**a**) 9:1-c-25-Y coupling agent: Y = 1.5, 1.0 and 0.5 from left to right; (**b**) 9:1-c-16.7-Y, coupling agent: Y = 1.5 and 1.0 from left to right; (**c**) 9:1-f-8.3-1.0 and -1.5 (26-1, 26-4); 9:1-f-16.7-1.5 (26-5); 9:1-f-25-1.0, -1.5 and -0.5 (26-3, 26-6, 26-7; (**d**) 9:1-c-8.3-1.0 and -1.5 (27-1, 27-4); (**e**) 9:1-c-16.7-1.0 and -1.5 (27-2, 27-5); (**f**) 3:4-c-8-1.0 and 3:4-c-16-1.0 (29-4, 29-5); (**g**) the sides of the tensile bars from (**a**) after 29 days, first six from the right; (**h**) the sides of the tensile bars from (**b**) after 29 days, the four in the middle; (**i**) surface of tensile bars from (d) after 289 days).

Water sorption in SBP containing PLA–PBS compounds is significantly affected by the size of the SBP particles. All composites containing the course SBP type showed crevices and cracks on the surface in a short time (days) independent from the polarity of the polymer matrix (Figure 1). Already after 1 day, the surface is roughened by swollen particles of SBP. During 14 days holes and crevices can be seen. Beginning around day 29, cracks on the highly filled composites (25% SBP; see Figure 1g) start to appear on the sides of the tensile dog-bone shaped test bars.

**Figure 2:**
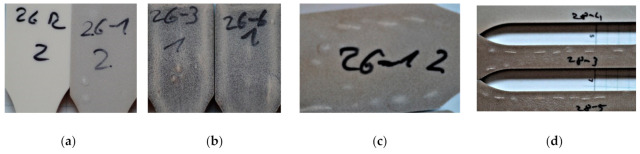
Tensile test bar immersed in water after 289 days; (**a**) 9:1-f-0-0 and 9:1-f-8.3-1.0 from left to right; (**b**) 9:1-f-25-1.0 and -1.5; (**c**) backside of (**a**) 9:1-f-8.3-1.0 showing a pattern of crevices on swollen particles; (**d**) 4:3-f-8-1.0 and 4:3-f-16-1.0 and 4:3-f-16-1.0 from top to down; the same pattern as in (**c**) can be seen in the composites with 16% SBP.

Composites with the fine SBP type do not show crevices and cracks on a short time scale. Their surface will remain smooth. However, they swell more obviously on a long time scale, changing the rectangular cross section to a slightly ellipsoid distorted shape. After several months, some crevices can be seen on the top of swollen fine particles (crevices on bump, Figure 2c,d and Table 2).

Surprisingly we noticed the same pattern on PLA rich 9:1-f-X-Y composites and on more PBS containing 4:3-f-X-Y composites. Furthermore, color changes due to varying degrees of leaching of solubles can be seen on the surfaces of test pieces that do not show any bumps or crevices (Figure 2b). A possible explanation is the influence of the injection molding parameters and the geometry of the mold leading to non-isotropical, but symmetric distribution of stresses along the cross section and therefore to anisotropic behavior of the internal stress caused by water absorption leading to such symmetrical patterns.

**Figure 3:**
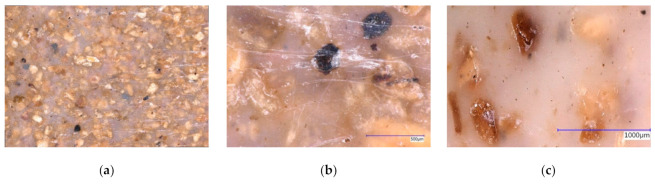
(**a**) 9:1-c-25-1.0, panorama mode, 7 × 10 mm; (**b**) 9:1-c-25-1.0 showing a burnt particle destroying the surface, magnification 200×, 3D mode; (**c**): 9:1-c-16.7-1.0 showing the rough surface, due to embedded particles, magnification 100×, 3D mode.

As it can be seen by microscopy (Figure 3), the coarse SBP particles are sometimes not fully embedded in the polymer matrix and often lay directly under a thin polymer skin. Different processes of sorption at early times might be responsible for the difference in the short time-scale behavior of the composites with the fine or coarse SBP. One explanation is the sorption of water of unbroken SBP cells at the surface or directly beneath it, leading to a specific osmotic pressure, which destroys the thin polymer layer.

On a long time scale, SBP-rich composites (coarse SBP-type) show embrittlement. This manifests practically by the sensitivity of the specimens to torque forces during drying the wet specimens. Meanwhile, the composites with the fine type, except the 3:4-f-16-1.0 composite, have sufficient strengths.

In particular, the embrittlement is most pronounced in the PBS-rich samples with 3:4-c-X-Y composition, even though the 4:3-c-X-Y composites show surface changes earlier in time. As shown in Section 4.2, the samples with PLA:PBS = 4:3 or 3:4 show a pronounced water absorption even in the reference sample without SBP. This embrittlement can partly be explained due to the morphology and composition of the matrix.

#### 4.1.2. Water Sorption Curves, Description

**Figure 4:**
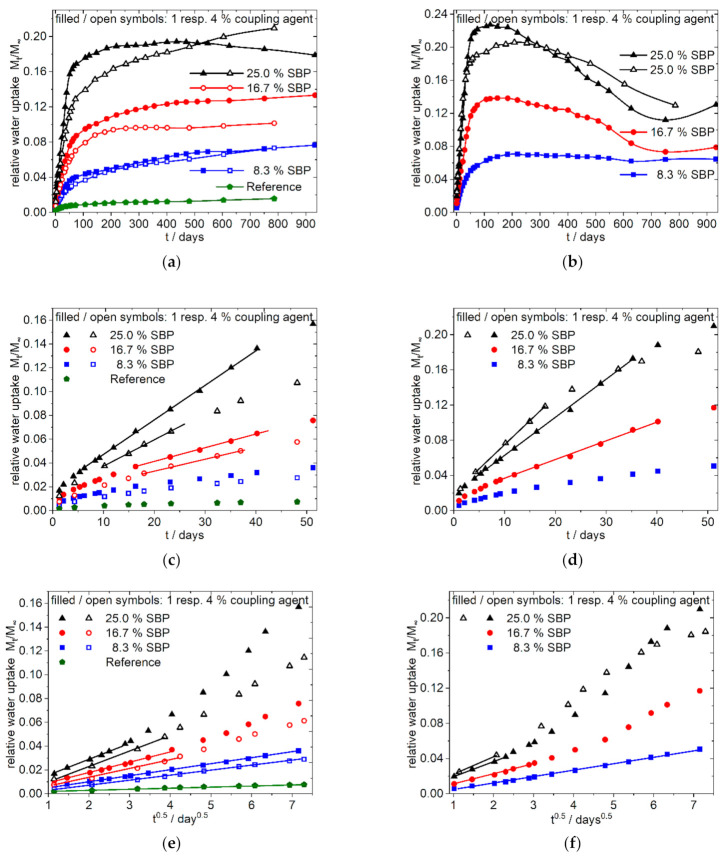
Water uptake of 9:1-f/c-8.3/16.7/25-1.0/4.0 Composites; fine type: (**a**,**c**,**e**); coarse type: (**b**,**d**,**f**); (**a**,**b**) overview, linear dependence from time; (**c**,**d**) enlargement of (**a**,**b**) for the first 50 days; (**e**,**f**) enlargement of the first 50 days of (**a**,**b**) showing the dependence of water uptake from the square root of time; filled and open symbols indicate: 1% resp. 4% of coupling agent per 10% of SBP.

**Figure 5:**
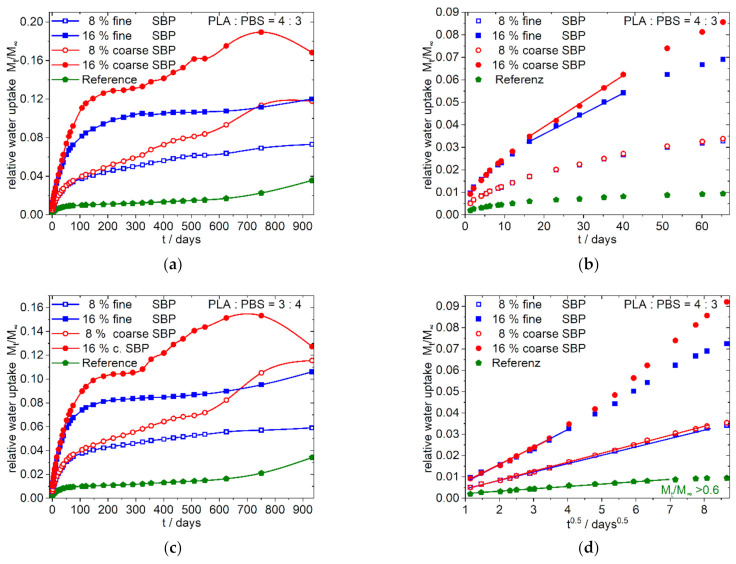
Water uptake of the 4:3/3:4-f/c-8/16-1.0 Composites; (**a**,**c**) overview, linear dependence from time; (**b**) enlargement of (**a**) for the first 70 days; (**d**) enlargement of (**a**) of the first 70 days showing the dependence of water uptake from the square root of time. The nearly identical shaped graphs for the 3:4-f/c-8/16-1.0 composites analogous to (**b**,**d**) is shown in the supplementary materials (Figure S5e,f).

The water sorption curves up to 940 days as well as the magnification of the curves at early times are shown in Figure 4 and Figure 5. The data of the water absorption measurements are available in the supplementary materials.

##### Overall Sorption Characteristics

The overall water sorption over a long time is dependent from the composition of the polymer matrix and the type of SBP. The polar 9:1-c-X-1.0 composites (Figure 4b,d,f) show a quick water uptake. The higher the amount of the coarse SBP, the faster the sorption reaches a maximum. With higher amounts of coarse SBP, no steady state is reached, instead the total mass decreases. As shown in Figure 1g,h the specimens show cracks within 1 month, and therefore the dissolution of solvable components like hemicelluloses and sugars seems to appear. Beginning with day 750, a new process with increasing water uptake starts. Contrary, the composites with the fine type (9:1-f-8.3/16.7-1.0, Figure 4a,c,e) show a permanent uptake of water up to 940 days for 8.33 and 16.67% SBP. Their shapes also indicate further processes on the medium time scale.

The less polar 4:3-f/c-8/16-1.0 composites (Figure 5) and the 3:4-f/c-8/16-1.0 composites (see supplementary materials) show nearly similar curve shapes. After almost reaching a plateau value in the range of 150–250 days, the water absorption for the composites with coarse SBP type increases again and passes through a maximum after about 2 to 2.5 years. The composites with fine SBP type, on the other hand, shows a steady slight increase (4:3/3:4-f-8-1.0) or, from approx. 600 days on, an intensified increase (4:3/3:4-f-16-1.0).

##### Sorption Characteristics at the Beginning

The data for the most polar composition 9:1-f/c-X-1.0 (Figure 4) indicate that at the beginning of the sorption process a purely diffusive behavior according to Equation (4) is present (Figure 4e,f), resulting in a straight line in the sorption plot against the square root of time. Deviations from this process occur earlier with higher amounts of SBP and for the coarser SBP type. Consequently, the higher the SBP content and the coarser the material, the earlier the onset of the linear dependency from time can be seen in the sorption plot against time (Figure 4c,d). Thus, in the case of 9:1-c-25-1.0 composite the case 2 type of sorption is most pronounced after 5 to 16 days while for the composite 9:1-c-8.3-1.0 the sorption is diffusion controlled over a longer time scale. Except the 9:1-f-8.3-1.0 composite, all 9:1-f/c-X-1.0 composites show sigmoidal shaped curves of the water sorption when the data are related to the square root of time over a longer time scale, indicating changes in the underlying processes (anomalous behavior) [20,22] (graphs are not shown, they can be generated from data in the supplementary materials).

In the less polar 4:3-f/c-X-1.0 composites, Figure 5a,b,d no major differences in water uptake up to day 75 can be seen for the composites with 8% fine or coarse SBP types. Moreover, in the case of 16% SBP, the deviations are less pronounced for the least polar 3:4-f/c-16-1.0 composites (not shown in Figure 5, see supplementary materials Figure S5e,f). Composites with 8% SBP show Fickian diffusion (case 1) over a longer time scale. Sigmoidal curves of water sorption have only been observed for the 4:3/3:4-c-16-1.0 composites, although this is only slightly pronounced for the 3:4-c-16-1.0 composite (graphs not shown, can be generated from data in the supplementary materials).

Generally, it can be stated that composites with the fine SBP type and/or with less SBP and/or with less polar matrices show a case I behavior at the beginning of the sorption. The characteristics will change to anomalous case 3 or case 2 sorption characteristics with more polar composites, more SBP and with coarser SBP types.

The addition of higher amounts of coupling agent slows down the water sorption velocity in the case of the 9:1-f-8.3/16.7/25-4.0 composites and the 9:1-c-25-4.0 composite, but it does not decrease the overall absorbed amount of water after a long time. This behavior, together with the mechanical characteristics given in the previous paper [5], also indicates the successful coupling of PLA chains to the SBP. Thus, the tightly adhering polymer chains make it difficult for the penetrating water to reach hydrophilic sites quickly but cannot prevent it in the long term.

### 4.2. Phenomenological Simulation of Sorption Curves

To gain better insight into the sorption process, we conducted a simulation study. Based on Equations (9)–(11) and the appearance of the sorption curves, we assumed (i) two diffusion and three relaxation modes or (ii) two diffusion, two relaxation and one exponential mode with the corresponding constants and the partial final water uptake coefficients *x_i_* (=*M_∞F,i;R,j_*; and *M_t,expo_^/^M_∞_*) belonging to the potential four to five processes (constraint: Σ*x_i_* = 1).

Furthermore, separate starting times are taken for the relaxation process. That is because it was noticed in the experiments, that swelling does not start with day one, except for the samples with higher amounts of the coarse SBP type. As fitting criteria, the minimized sum of the errors in calculated and experimental values was chosen (Equation (17)):

(17) $$\mathrm{error}=\underset{i}{\sum }{\left({\left(M_{exp}-M_{calc}\right)}^{2}\right)}^{0.5}$$

Additionally, each single error should be less than about 0.01 (<1% of the normalized data), as the relative standard deviations of the measurement data are often less than one percent, especially for composites with the fine SBP type.

**Figure 6:**
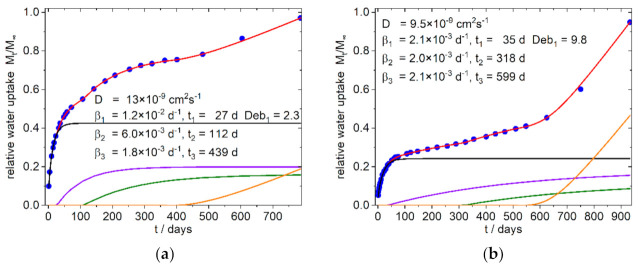
Simulation of water uptake of the reference Composites: (**a**) 9:1-0-0; (**b**) 4:3-0-0; Blue dots: experimental normalized values, red line: simulation, black line: diffusion part of water uptake, purple and olive line: part of water uptake from the relaxation processes 1 and 2, orange line part of water uptake from the exponential growth of water uptake. The nearly identical shaped graph for the 3:4-0-0 composite is shown in the supplementary materials (Figure S6c).

**Figure 7:**
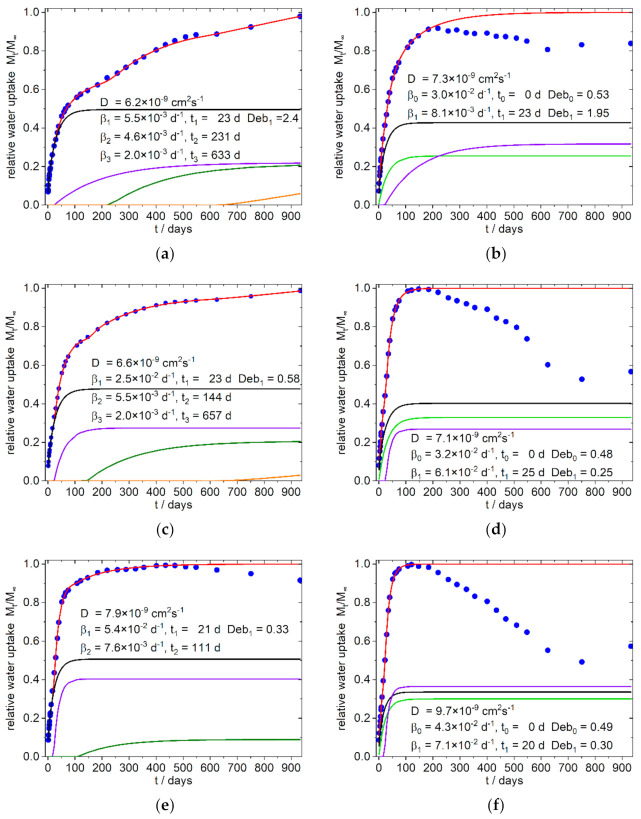
Simulation of water uptake of the 9:1-f/c-X Composites with X = 8.3 (**a**,**b**), 16.7 (**c**,**d**), 25 (**e**,**f**); mass loss in (**b**,**d**,**f**) are presumably due to extracted hemicelluloses and soluble low molecular weight carbohydrates from the SBP particles. Blue dots: experimental normalized values, red line: simulation, black line: diffusion part of water uptake, green line: part of water uptake from the relaxation process with starts with t = 0, purple and olive line: part of water uptake from the relaxation processes 1 and 2, orange line part of water uptake from the exponential growth of water uptake.

**Figure 8:**
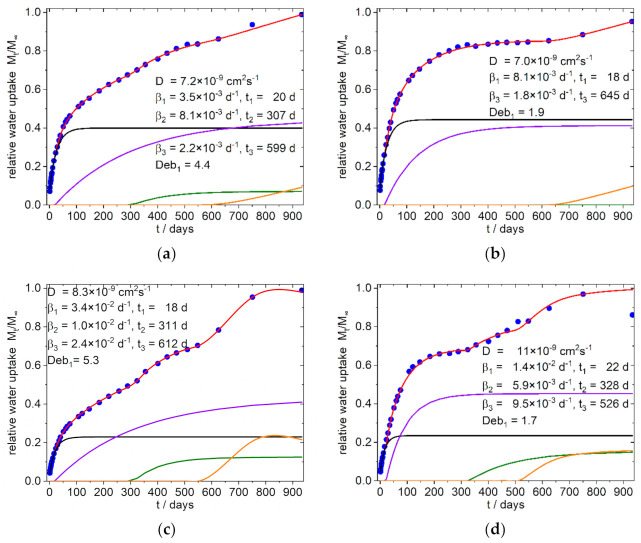
Simulation of water uptake of the 4:3-f/c-X Composites; (**a**) 4:3-f-8; (**b**) 4:3-f-16; (**c**) 4:3-c-8; (**d**) 4:3-c-16; the nearly identical shaped graphs for the corresponding 3:4-f/c-X composites are shown in the supplementary materials (Figure S8e,f,g,h); Blue dots: experimental normalized values, red line: simulation, black line: diffusion part of water uptake, purple and olive line: part of water uptake from the relaxation processes 1 and 2, orange line: part of water uptake from the exponential growth of water uptake or from the relaxation processes (see text).

The simulation was conducted with the solver plug-in of the Excel^®^-Software from Microsoft (version 2016). Due to the highly parameterized approach, several solutions are to be expected. We therefore took into account that for the whole system consistent solution should result in order to interpret the data phenomenological in their entirety. Simulations, which results in nearly identical diffusion coefficients *D*_1_ and *D*_2_, as well as results which showed only a small contribution to the total absorption, were excluded and the simulation was conducted again with one diffusion term. The same procedure was applied, if in the terms describing the relaxation the time offset and the frequency *β* were close together. As indicated by Equation (11), the saturation level of sorption is needed for the calculation. In cases where the curves did not show a clear limit the highest measured arbitrary value was taken as a starting value. Simulation was done several times by varying the starting values to find a first minimum of the sum of error. Next, the same process was conducted with the best fit again, by varying the found numbers by some percent. At last, the saturation level was included in the parameter set, all parameters were disturbed by some percent again, and simulation was run once again for several times. Due to the seasonal variation in temperature during the sorption process an underfitting of the summer time datapoints was chosen instead of under- and overfitting of datapoints. The temporal evolution of the sorption and its simulation are shown for the references in Figure 6 and for the PLA–PBS–SBP composites in Figure 7 and Figure 8. The obtained data from the simulation of the sorption curves are summarized in Table 3 and Table 4. All composites selected for simulation contained 1% PLA-g-MAH per 10% SBP. Therefore, the index Y for this content in the sample designation is omitted in the further discussion.

Surprisingly it was found that in all simulations only one Fickian type diffusion was sufficient to describe the system, which was accompanied by two relaxation sorption modes in the first 400 days (except sample 8 b, with one relaxation mode). These phases are followed by an exponential growth in water uptake, which starts after about 400–600 days in the samples with fine SBP type, as well as in the samples with the compositions without SBP (Figure 6). In contrast, the 4:3/3:4-c-X composites, at this stage show a water uptake that can better be described with a relaxation model. The 9:1-c-X composites are destructured at an early stage and lose mass after about 100 days. Surprisingly, however, after about 600–750 days there is an increase in the mass of these samples, which by this time are already mechanically labile and heavily destructured at the surface.

#### 4.2.1. 9:1-f/c/-X Composites

In the polar PLA rich 9:1-f-X composites (Figure 7a,c,e) the diffusion constants as well the first relaxation frequency (*β*_1_) increase with higher fine SBP content. The relative amount of water uptake also increases for the first relaxation mode (purple line in Figure 7a,c,e) and the onset of the second relaxation mode decreases with higher fine SBP content (olive line in Figure 7a,c,e).

For the 9:1-c-X composites we fixed the starting time for the first relaxation mode (indicated with *β*_0_ in Table 3, bright green line in Figure 7b,d,f) to zero because the deviations from linearity in Figure 4f (water uptake versus t^0.5^) start early, indicating an additional early non diffusive sorption. The relative water absorption per percent SBP of this relaxation *β*_0_ is lower than the relative water absorption per percent SBP of the first relaxation modes (*β*_1_, starting later in time) in the 4:3/3:4-c-X composites. The sum of the relative water uptakes per percent SBP of the first two relaxation modes, *β*_0_ and *β*_1_ (see Table 4) in the 9:1-c-X composites, are of similar order of magnitude as the relative water uptake of the first relaxation *β*_1_ in the 4:3/3:4-c-X4 composites. Therefore, it can be deduced that this relaxation *β*_0_ and *β*_1_ are probably due to the same causes, but the water uptake takes place by two different routes.

Increasing the amount of coarse SBP will also increase the relaxation frequency *β*_1_, as in the case of the fine type, and decrease the relative amount of diffusion to the sorption, but has no great effect on the onset. A significant jump in the magnitude of the first characteristic relaxation frequency between the samples with 8.3 and 16.7 and 25% coarse SBP, respectively, can be seen in the data from the simulations. A similar increase is also observed for the samples with higher PBS amounts. On the long time scale, an exponential increase in water absorption is indicated for the fine SBP type (Figure 7a,c, orange line). This can also be seen in the reference sample (Figure 6a) and in the 4:3/3:4-f-X composites (Figure 8a,b, and supplementary materials).

The diffusion coefficients are slightly lower than in the reference with chalk instead of SBP (*D* = 13 × 10^−9^ cm^2^s^−1^) which may be due to sorption sites with better binding properties that will bind parts of the diffusing water molecules almost irreversibly, so that the overall probability of a water molecule migrating further decreases (see also the discussion in [20,21]). However, the deviations are not so large as to be significant. The highly parameterized system allows simulation solutions with closely spaced sum errors. By and large, the composites presented here behave as expected with diffusion coefficients in the range of 10^−8^ cm^2^s^−1^ [28,29]. Therefore, as we tested the sorption in water and not in soil with varying content on water or humidity, the time-scale of the processes presented here should be the lower scale for such processes in the environment. Aging processes through absorption and desorption are not taken into account here and may alter the processes.

From our observations and simulations, it can be concluded, that in the 9:1-f-X composites, the sorption characteristic can be designed to some extent by choosing an appropriate SBP content. Higher proportions of fine SBP of more than 25% should shift the sorption characteristic to that of the composites with the coarse SBP type with early mass loss. Composites with coarse SBP type are characterized by the early effects of SBP particles near the surface. In this case, it is to be expected, that at higher content of SBP, the surface is further destroyed and underlying layers quickly become accessible. The material should therefore disintegrate in short time scales with sufficient water supply, which in turn promotes biodegradability.

#### 4.2.2. 4:3/3:4-f/c-Xcomposites

In the less polar 4:3/3:4-f/c-X composites, (Figure 8 and Figure S8 and Table 3) the diffusion coefficients are in the same range as in the higher polar 9:1-f/c-X composites.

The beginning of the first relaxation happens after the same delay (20–25 days), but the relaxation frequencies *β*_1_ are somewhat reduced. Contrary to the more polar 9:1-f/c-X composites, the second relaxation *β*_2_ is delayed but the relaxation frequencies *β*_2_ are in the same order of magnitude. The coarse SBP particles have a greater effect on this relaxation as can be seen by the greater relative water uptake per percent SBP (Table 4). Furthermore, the delay of the relaxation 2 and 3 in the 4:3/3:4-c-16 composites are closer together than in the 4:3/3:4-c-8 composites, indicating a beginning convergence of these relaxations. While the water absorption of the 4:3/3:4-c-8 composites appears to reach saturation between 600 and 900 days, the 4:3/3:4-c-16 composites show a mass decrease from about 750 days on. Since exponential water uptake already occurs in these time periods in the 4:3/3:4-f-8 composites, we assume that the observed saturation of water uptake in the 4:3/3:4-c-8 composites is only apparent. It might rather result from two simultaneous antagonistic processes: one being the accelerated water uptake due to hydrolysis and the other being the disintegration and dissolution of the composite.

## 5. Conclusions

Water uptake in composites of SBP and the bio-based polyesters PLA and PBS can be described using a model with one diffusion term and two or three relaxation terms, or two relaxation terms and one exponential term, respectively. The analysis of the time dependent sorption at the beginning shows clearly the presence of a relaxation mode at early stages of the sorption. In the case of the polar 9:1-f/c-X composites, the analysis of early sorption clearly indicates the coupling of diffusion and relaxation processes.

The diffusion coefficients are in the expected range of 10^−8^ cm^−2^s^−1^ and they do not significantly change in the presence of the SBP. Since the first relaxation *β*_1_ also occurs in SBP-free composites and generally starts after about 20–30 days, we interpret it as the softening effect of the diffusing water followed by the morphological rearrangement of the polymeric chains on a molecular level (sub-nano-scale to nano-scale) with the inclusion of additional water at then accessible sites. The presence of coarse SBP or larger amounts of fine SBP accelerates this process. With increasing amounts of SPB, the relative proportion of adsorbed water also increases, especially in the less polar PBS-rich composites. We interpret this with the provision of transport routes for water uptake by the SBP. Additionally, some SBP constituents itself rearrange and absorb greater amounts of water due to their high content on polar binding sites, especially the highly polar carboxyl groups in the pectinic moieties.

We have no clear understanding of the second relaxation on a medium time scale (~300–500 days) since we also detected this phenomenon in SBP free, only mineral filled composites. For the SBP containing composites, the assumption of rearrangement of the amorphous OH group-containing domains of the carbohydrates in SBP under water uptake is reasonable, but it does not explain the different relative water uptakes per percent SBP in the fine and coarse SBP type composites. We assume that osmotic processes may play a role here. This is supported by the relative water absorption of the third relaxation of composites with coarse SBP particles, containing whole cells, compared to composites with fine particles containing only fragments of cells. For the SBP-free composites a rearrangement of the morphology in chain dimensions (nano-scale) is assumed. The type of the “third” relaxation in the 4:3/3:4-c-X composites as well as the influence of SBP on the disintegration and biodegradation of biodegradable polyesters will have to be analyzed in more detailed future research.

## Figures and Tables

**Table 1:** Composition of the PLA–PBS–SBP composites in mass percent; “f” or “c” describes the type of SBP, fine or coarse, resp., “X” describes the amount of SBP in wt.% and “Y” the amount of coupling agent in wt.% added per 10% of SBP. The coupling agent amount was added additionally. Sum of PLA, PBS, SBP and mineral filler = 100%.

Sample Designation	PLA:PBS	PLA	PBS	SBP, Fine (f) or Coarse (c) Type	Chalk	Talc	Coupling Agent
	Matrix composition			X			Y
9:1-f-X-Y	9:1	67.5	7.5	0/8.3/16.7/25	25/16.7/8.3/0	0	0.5/1.0/1.5/4
9:1-c-X-Y				0/8.3/16.7/25	25/16.7/8.3/0	0	0.5/1.0/1.5/4
4:3-f-X-Y	4:3	48	36	0/8/16	0	16/8/0	1.0/1.5
4:3-c-X-Y				0/8/16	0	16/8/0	1.0/1.5
3:4-f-X-Y	3:4	36	48	0/8/16	0	16/8/0	1.0/1.5
3:4-c-X-Y				0/8/16	0	16/8/0	1.0/1.5

**Table 2:** Observations during weighing of test specimens from water sorption; all samples with coarse SBP type show open bumps at the surfaces within 1 day; embrittlement: test specimen will break when torque is applied during drying of the wet specimen.

Sample Designation	Day	Observation	Day	Observation	Day	Observation
9:1-0-0-0.0						
9:1-f-8.3-1.0	257	bump	289	bump with crevice		
9:1-f-16.7-1.0	257	bump with crevices				
9:1-f-25-1.0	219	bump with crevices				
9:1-c-8.3-1.0	12	crevices/cracks	51	cracks	510	edge breaking
9:1-c-16.7-1.0	12	crevices/cracks	29	cracks		
9:1-c-25-1.0	12	crevices/cracks	29	cracks	751	embrittlement
4:3-0-0-0.0					933	bump
4:3-f-8-1.0			625	bump	933	crevices
4:3-f-16-1.0	219–289	bump with crevices	625	crevices	933	crevices
4:3-c-8-1.0	355	crevices/cracks	401	cracks	625	long cracks
4:3-c-16-1.0	107	crack	401	long crack	625–933	embrittl. delamination
3:4-0-0-0.0					933	bump
3:4-f-8-1.0	355	beginning bump			933	crevices
3:4-f-16-1.0	355–455	bump	625	crevices	751–933	embrittlement
3:4-c-8-1.0			625	crevices	751	embrittlement
3:4-c-16-1.0	147	crevices	184	crevices/cracks	751	embrittlement

**Table 3:** Characteristic parameters of diffusion and relaxation phenomena from simulation of PLA–PBS–SBP composites.

Sample Designation	Diffusion-Coefficient 10^−9^ cm^2^s^−1^	Relaxation/Expon. Growth				Frequency Coefficient Beta/ 10^−3^ d^−1^					Deb		∅ Error Per DataPoint
		Day				Relaxation				Growth			
		d_0_	d_1_	d_2_	d_3_	*β*_0_	*β*_1_	*β*_2_	*β*_3_	*β*_3_	*β*_0_	*β*_1_	
9:1-0-0	13		27	112	438		12	6.0		1.8		2.3	0.0022
9:1-f-8.3	6.2		23	231	633		5.5	4.6		2.0		2.4	0.0051
9:1-f-16.7	6.6		23	144	657		25	5.5		2.0		0.58	0.0034
9:1-f-25	7.9		21	111			54	7.6				0.33	0.0071
9:1-c-8.3	7.3	0	23			30	8.1				0.53	2.0	0.0032
9:1-c-16.7	7.1	0	26			32	61				0.48	0.25	0.0036
9:1-c-25	9.7	0	20			43	71				0.49	0.30	0.0069
4:3-0-0	9.5		35	318	599		2.1	2.0		2.1		9.8	0.0032
4:3-f-8	7.2		20	307	599		3.5	8.1		2.2		4.4	0.0037
4:3-f-16	7.0		18		645		8.1			1.8		1.9	0.0035
4:3-c-8	8.3		18	311	612		3.4	10				5.3	0.0020
4:3-c-16	11		22	328	526		14	5.9				1.7	0.0060
3:4-0-0	11		20	293	600		2.4	1.4		2.3		9.8	0.0042
3:4-f-8	6.5		23	305	405		3.4	4.2		1.7		4.1	0.0030
3:4-f-16	5.9		19	419	582		11	4.2		1.9		1.2	0.0023
3:4-c-8	8.1		25	285	597		4.0	4.3	8.4			4.4	0.0015
3:4-c-16	18		18	318	408		15	12	6.3			2.5	0.0038

**Table 4:** Water sorption parameters from simulation of PLA–PBS–SBP composites.

Sample Designation	Satu-Ration	Mass Fraction ^1^						Relative Sorption Per Percent SBP ^1^						
		X_D_	X_0_	X_R1_	X_R2_	X_R3_	X_e.gro._	Sum	D	R_0_	R1	R2	R3	e.gro.
9:1-0-0	1.61%	0.43		0.20	0.16		0.21							
9:1-f-8.3	7.81%	0.50		0.22	0.22		0.07	0.94	0.46		0.21	0.20		0.07
9:1-f-16.7	13.5%	0.48		0.28	0.21		0.04	0.81	0.39		0.22	0.17		0.03
9:1-f-25	19.5%	0.51		0.40	0.09			0.78	0.40		0.32	0.07		
9:1-c-8.3	7.69%	0.43	0.26	0.32				0.92	0.39	0.24	0.29			
9:1-c-16.7	13.9%	0.40	0.33	0.27				0.83	0.34	0.27	0.22			
9:1-c-25	22.8%	0.34	0.30	0.36				0.91	0.31	0.27	0.33			
4:3-0-0	3.76%	0.24		0.18	0.12		0.45							
4:3-f-8	7.38%	0.40		0.44	0.07		0.09	0.92	0.37		0.41	0.07		0.08
4:3-f-16	12.6%	0.44		0.41			0.15	0.79	0.35		0.32			0.11
4:3-c-8	11.9%	0.23		0.43	0.13	0.22		1.49	0.34		0.64	0.19	0.32	
4:3-c-16	19.5%	0.24		0.45	0.15	0.16		1.22	0.29		0.55	0.19	0.20	
3:4-0-0	3.61%	0.25		0.15	0.19		0.42							
3:4-f-8	5.95%	0.56		0.35	0.06		0.03	0.74	0.42		0.26	0.05		0.02
3:4-f-16	10.7%	0.58		0.22	0.05		0.15	0.67	0.39		0.15	0.04		0.10
3:4-c-8	12.0%	0.26		0.30	0.11	0.33		1.50	0.39		0.45	0.17	0.49	
3:4-c-16	15.6%	0.24		0.45	0.15	0.16		0.98	0.23		0.44	0.15	0.16	

^1^ sum (column 9): saturation (column 2) divided by the SBP content in %; column 10–15: saturation (column 2) multiplied with the partial water uptake (column 3–8) and divided by the SBP content in %.

## Data Availability

For details of the data used in this paper and for details of programming the Excel based Solver-Add-In as well as for additionally graphs, see the supplementary materials.

## Supplementary Materials

The following are available online at https://www.mdpi.com/article/10.3390/polym13203558/s1, Figures S5, S6 and S8: Figure_S5_S6_S8.pdf, measured data of water sorption: water_sorption_data_(US).xlsx, Excel™ based simulation sheet for the sorption processes: simulation_sheet_(US).xslx (the Solver-Add-In in Excel must be activated to run the simulation).
