# Supplementary material for: Poly(lactic acid)–Poly(butylene succinate)–Sugar Beet Pulp Composites; Part II: Water Absorption Characteristics with Fine and Coarse Sugar Beet Pulp Particles; A Phenomenological Investigation
Source: Polymers (Basel). 2021 Oct 15;13(20):3558. doi: 10.3390/polym13203558 (PMC8541516; doi:10.3390/polym13203558)
Supplement: Supplementary file 1 [file polymers-13-03558-s001.zip › Figure_S5_S6_S8.pdf]

Supporting Informations:

Poly(lactic acid)–Poly(butylene succinate)–Sugar Beet Pulp Composites; Part II: Water Absorption Characteristics with Fine and Coarse Sugar Beet Pulp Particles; a phenomenological investigation

Additionally graphs not shown in the main text:

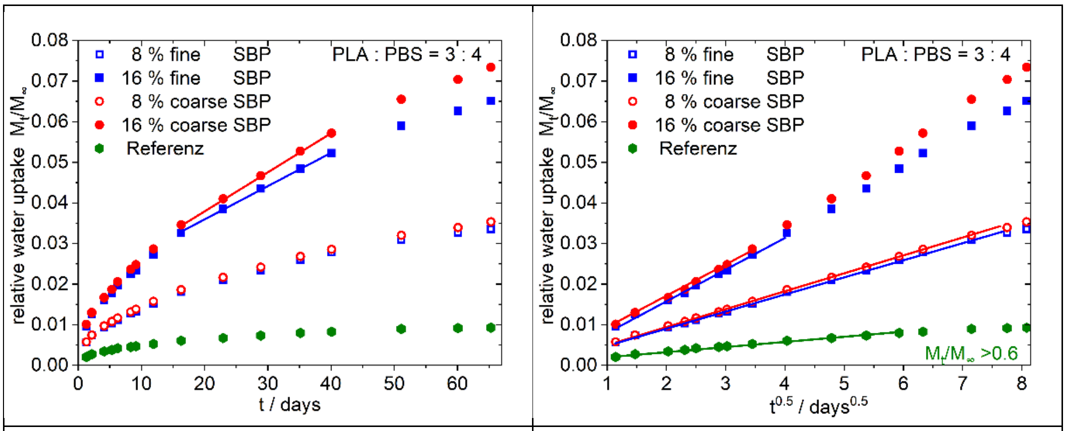

(e) (f)

Figure S5: water uptake of the 3:4-f/c-8/16-1.0 Composites; (**e**) enlargement of fig 5c in the article for the first 70 days, linear dependence from time; (**f**) enlargement of fig. 5c in the article of the first 70 days showing the dependence of water uptake from the square root of time.

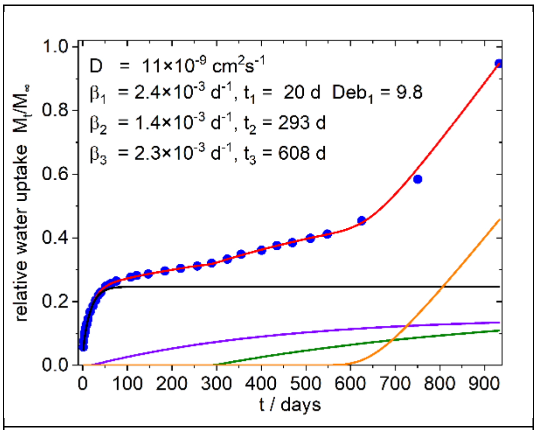

(c)

Figure S6: Simulation of water uptake of the 3:4-0-0 Composites: (**c**) 3:4-0-0; Blue dots: experimental normalized values, red line: simulation, black line: diffusion part of water uptake, purple and olive line: part of water uptake from the relaxation processes 1 and 2, orange line part of water uptake from the exponential growth of water uptake.

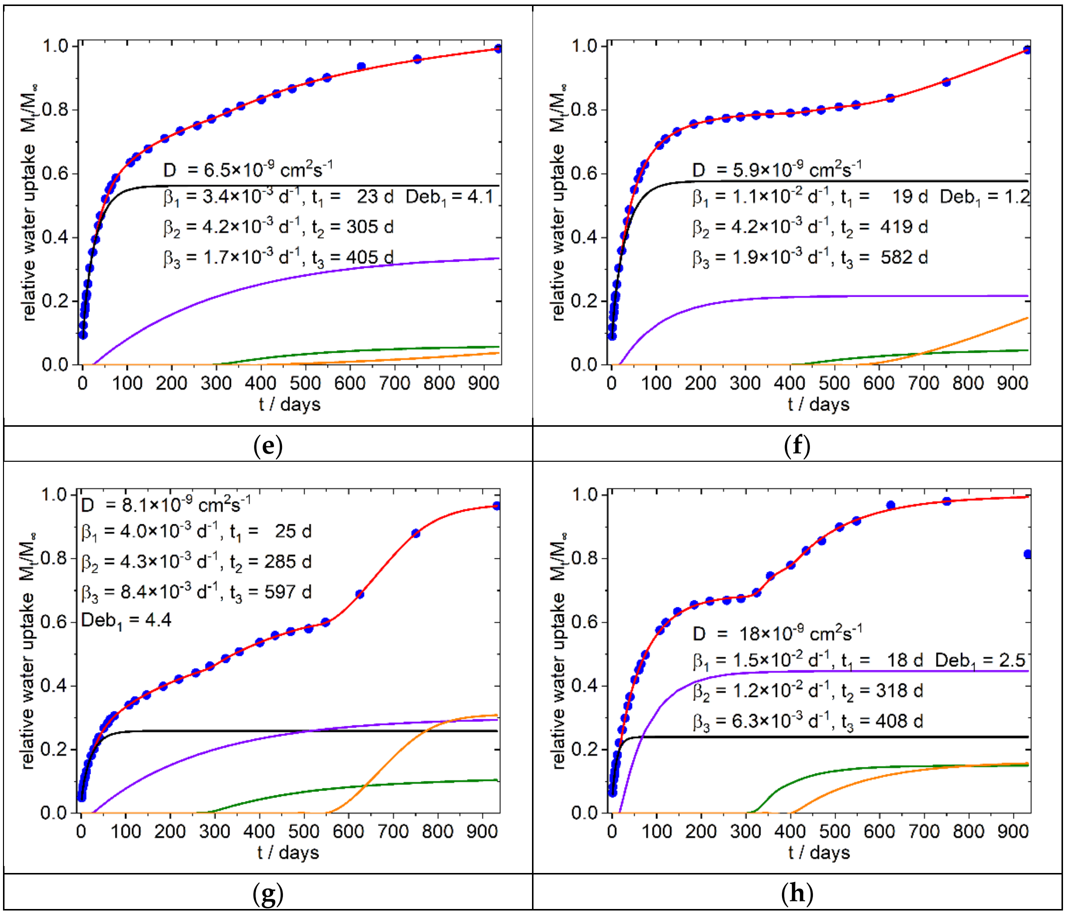

Figure S8: Simulation of water uptake of the 3:4-f/c-X Composites; (**e**) 3:4-f-8; (**f**) 3:4-f-16; (**g**) 3:4-c-8; (**h**) 3:4-c-16; Blue dots: experimental normalized values, red line: simulation, black line: diffusion part of water uptake, purple and olive line: part of water uptake from the relaxation processes 1 and 2, orange line part of water uptake from the exponential growth of water uptake or from the relaxation processes(see text).
